# Sex-Biased Temporal Gene Expression in Male and Female Floral Buds of Seabuckthorn (*Hippophae rhamnoides*)

**DOI:** 10.1371/journal.pone.0124890

**Published:** 2015-04-27

**Authors:** Aseem Chawla, Tsering Stobdan, Ravi B. Srivastava, Varun Jaiswal, Rajinder S. Chauhan, Anil Kant

**Affiliations:** 1 Department of Biotechnology and Bioinformatics, Jaypee University of Information Technology, Waknaghat, Solan, India; 2 Defence Institute of High Altitude Research, Defence R & D Organisation, Leh, Jammu, and Kashmir, India; University of Perugia, ITALY

## Abstract

Seabuckthorn is an economically important dioecious plant in which mechanism of sex determination is unknown. The study was conducted to identify seabuckthorn homologous genes involved in floral development which may have role in sex determination. Forty four putative Genes involved in sex determination (GISD) reported in model plants were shortlisted from literature survey, and twenty nine seabuckthorn homologous sequences were identified from available seabuckthorn genomic resources. Of these, 21 genes were found to differentially express in either male or female flower bud stages. *HrCRY2* was significantly expressed in female flower buds only while *HrCO* had significant expression in male flowers only. Among the three male and female floral development stages (FDS), male stage II had significant expression of most of the GISD. Information on these sex-specific expressed genes will help in elucidating sex determination mechanism in seabuckthorn.

## Introduction

*Hippophae rhamnoides* commonly known as seabuckthorn belongs to the family Elaeagnaceae. Seabuckthorn berries are among the most nutritious and vitamin-rich fruits found in the plant kingdom. In general, the flesh of berries contains a diverse complex of vitamins, mineral substances such as sodium salts, potassium, calcium, carbohydrates, proteins, sugars and amino acids. [1, 2]. Moreover, the oil from the sea buckthorn berry contains on average 35% of the rare and valuable palmitoleic acid (16:1n-7; omega-7 series fatty acid) [3]. Seabuckthorn has a great potential for researchers in the field of biotechnology, neutraceutical and environmental sciences [4]. Various products had been developed from the berries of seabuckthorn such as oil, juice, alcoholic beverages, candies, ice-cream, tea, jam and biscuits. [3]. Thus the demand of seabuckthorn berries has increased in past few years due to their increased use in commercial products [5]. This increase in demand warrants its intensive cultivation, instead of just collection from wild resources and requires genetic improvement to increase its productivity and quality.

For development of superior seabuckthorn, breeding projects target both females and male cultivars [3]. Moreover, the objectives for breeding male and female plants vary, since there are extra quality criteria to be met in female plants, as berries occur on female plants only [3]. The success of the breeding program in dioecious plants depends upon early identification of progeny's gender. Unfortunately, gender of seabuckthorn seedlings cannot be determined morphologically until flowering, which usually occurs after 3–4 years in the field [6]. This represents a serious problem for plant breeders who are forced retain large number of male for several years. Much of the work and money could be saved if large proportion of the males could be discarded at an early stage in evaluation process.

In dioecious plants gender determination is regulated at genetic level by X/Y chromosome system [7]. Many molecular marker based studies like RAPD, SSR, ISSR, SCAR etc. were conducted for past several years for gender identification in seabuckthorn and molecular markers can distinguish male and female plants. [8–12]. However, none of the marker based studies in seabuckthorn related the markers with the mechanism governing sex determination. Therefore the mechanism governing the sex determination in seabuckthorn still remains unknown [3].

Differences between male and female plants are primarily detected in reproductive organs, which occur through differential growth, repression or abortion of sex organs in unisexual flowers [13, 14]. Various category of genes like floral mersitem identity genes, floral organ identity genes and flowering time genes play a major role as Genes involved in sex determination (GISD) in development of unisexual flowers [15, 16]. In case of *Thalictrum dioicum*, floral organ identity genes were differentially expressed in early development stages of male and female flowers. This led to the conclusion that regulation of these homeotic genes resulted in gender determination in this species [17]. Also the role of MADS box homeotic genes was analysed in male and female flowers of Hop (*Humulus lupulus*). Northern hybridisation in *H*. *lupulus* showed that M1 (*DEFICIENS* homologue) and M2 (*Petunia FLORAL BINDING PROTEIN 2* homologue) transcripts were present in the early stages of floral development of both sexes, but at later stages, expression of both genes increased in male flowers and decreased in female flowers [18, 19]. Moreover, apart from floral regulatory genes, sex determination is also dependent upon the regulatory networks which alter sex expression based on environmental cues such as photoperiod and temperature [20].

The genetic control of sex determination is well-kown in several model plant systems like *Silene latifolia* [21–23], *Cucumis sativus* [24–26], *Salix* [27, 28], etc. Moreover, molecular and genetic studies showed that the underlying mechanisms controlling flower development are largely conserved in distantly related dicotyledonous plant species [29]. Thus, genomic resources generated from these model plants could be used to identify the potential GISD in seabuckthorn. A possible strategy to identify genes essential in a development process is to screen mRNAs that are present in one sample and absent (or rare) in other ones [30]. In order to identify mRNA transcripts involved in sex determination in dioecious plants like *S*. *latifolia*, *Rumex acetosa*, and *Actinidia chinensis*,. different spatial and temporal development stages of flower were used [31–33]. Numerous flowering genes like *APETALA 2*, *CLAVATA 1* and *SEPTALA 3* showed differential expression among male and female flowers of plants like *Z*. *mays*, *S*. *latifolia*, *A*. *Officinalis* [34–36], which indicated their role in sex determination in the above mentioned plants. Thus for identification of potential seabuckthorn GISD, differential expression of known flowering genes was analysed using quantitative Real Time PCR (qRT-PCR) in three temporal Floral Development Stages (FDS) of both male and female seabuckthorn flowers.

## Material and Methods

### Plant material, RNA extraction and cDNA synthesis

**Fig 1:**
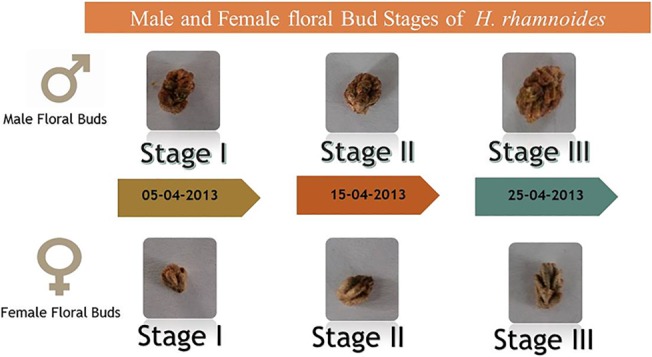
Temporal male and female floral bud development stages in seabuckthorn.

The flower buds of *Hippophae rhamnoides* collected from Defence Institute of High Altitude Research (DIHAR), J&K, India (Geographic Coordinates—34°08’ 236” N, 77° 34’ 345” E) were used in this investigation (Permission granted by Director, DIHAR, Leh, Jammu and Kashmir, India). Three different samples of floral buds for current study were collected on the basis of phenological observations on flowering of seabuckthorn in the region of study. Flower buds start developing from the month April and flowers open in the start of May to mid-May. The flower bud samples were collected in the month of April at ten days interval, starting from dormant winter bud to when buds are about to open. This is period when female and male reproductive tissues are formed in the flower buds. Flower buds were immediately frozen in liquid nitrogen and were stored at -80 °C till further use. Male and female flower bud stages were designated as Male Stage I (MST I), Male Stage II (MST II), Male Stage III (MST III) and Female Stage I (FST I), Female Stage II (FST II) and Female Stage III (FST III) respectively as shown in Fig 1. RNA was extracted from flower buds using Bangalore Genei Plant Total RNA extraction kit as per manufacturer instructions. RNA concentration was estimated by U.V. spectrophotometry and integrity was confirmed by electrophoresing samples on a 1.2% denaturing agarose gel. First strand of cDNA was synthesised from 1 μg of total RNA using Verso cDNA Kit (Thermo Scientific). The quality of cDNA was tested by amplifying 26S gene fragment using 26S primers (S1 Table) under following amplification conditions (95°C for 4 min and then 35 cycles at 95°C for 30 s, 55.5°C for 30 s and 72°C for 50 s) and products were electrophoresed in 1.8% agarose gel.

### Identification of seabuckthorn homologues of potential GISD and phylogenetic analysis

A literature survey was undertaken to short list genes involved in flower development of *Arabidopsis* which could be potential candidates for sex determination in seabuckthorn (Table 1). Nucleotide sequences of floral regulatory genes well-characterized in plants like *Silene latifolia*, *Arabidopsis thaliana*, *Vitis vinifera*, *Cucumis sativa*, etc. were downloaded from NCBI Genbank database in FASTA format (S1 File). The sequence data was manually curated and redundant sequences of the same species were discarded. Quality trimmed and filtered nucleotide sequences of seabuckthorn were retrieved from seed [37], root and leaf [38] transcriptome (NCBI Accession No.SRX118240, SRX131619 and SRX131618 respectively) and ESTs from NCBI EST database. A series of BLASTN analyses with default parameters identified broadly conserved sequences of potential GISD from seabuckthorn genomic resources showing syntenic relationship with known GISD (Table 2 and S2 File). BLASTN reports were analysed manually and the sequences (showing similarity with known GISD sequences) having e-value greater than 10^–4^ and query coverage less than 100bp were discarded. Homologous sequences of GISD having the lowest e-value were chosen for validation through qRT-PCR (Table 2). To further confirm the identity of the seabuckthorn sequences, domains and repeats were identified within the GISD sequences. Nucleotide sequences of putative seabuckthorn GISD were translated to amino acid coding sequences (S3 File) using ExPASy translate tool (http://web.expasy.org/translate/). The sequences with longest open reading frame were used for repeats, domain and protein family identification using EBI Interpro server (http://www.ebi.ac.uk/interpro/). For Phylogenetic reconstruction of potential GISD in seabuckthorn, protein sequences of known GISD characterized in model plant species were downloaded from NCBI Genbank database (S4 File). The alignment of the sequences was done with the help of CLUSTALX [39] and the final tree was constructed using MEGA 6 (Molecular Evolutionary Genetics Analysis 6.0) software [40].

**Table 1:** List of potential genes involved in sex determination in seabuckthorn.

S. No.	Gene Name	Function in flower development	References
1	*APETALA1* (*AP1*) / *SQUAMOSA* (*SQUA*)	Promotes sepal Differentiation, Supresses axillary bud initiation, required in secondary whorl development (CLASS A MADS box gene)	[42–44]
2	*APETALA2* (*AP2*)	Sepal identity (CLASS A MADS box gene)	[45]
3	*APETALA3* (*AP3*) / *DEFICIENS* (*DEF*)	Petal Identity in second whorl of flower, stamen identity in third whorl of flower (CLASS B MADS box gene)	[45]
4	*AGAMOUS* (*AG*) / *PLENA* (*PLE*)	Stamen Identity in third whorl of flower, carpel identity in fourth whorl of flower. (CLASS C MADS Box gene)	[46, 47]
5	*CAULIFLOWER* (*CAL*)	Floral meristem identity gene.	[29, 48]
6	*CRAB'S CLAW* (*CRC*)	Regulates carpel development	[49]
7	*CLAVATA1* (*CLV 1*)	Encodes putative receptor kinase which controls shoot and floral meristem size	[50]
8	*CONSTANS* (*CO*)	Regulates flowering time in response to day length	[51]
9	*CRYPTOCHROME1* (*CRY1*)	Blue ultraviolet A receptors. Regulates flowering time	[52]
10	*CRYPTOCHROME2* (*CRY2*)	Blue ultraviolet A receptors. Regulates flowering time	[52]
11	*EARLY FLOWERING 1* (*ELF1*)	Regulates FLC. Mutations in *EF1* results in suppression of FLC-mediated delay of flowering and causes early flowering in non-inductive photoperiods independently of FLC	[53]
12	*FILAMENTOUS FLOWER* (*FIL*)	Floral organ polarity	[54]
13	*JAGGED* (*JAG*)	Involved in the formation of lateral organs. *JAG* promotes distal petal development by suppressing premature cell-cycle arrest.	[55]
14	*KNUCKLESS* (*KNU*)	It encodes a C2H2 zinc-finger protein that regulates development of basal pattern elements along the proximo-distal axis of the developing gynoecium.	[56]
15	*LEAFY* (*LFY*) /*FLORICAULA* (*FLO*)	Promotes the expression of meristem identity *AP1*. Together with other co factors it activates the floral organ identity genes like *AP3* and *AGM*.	[57, 58]
16	*NOZZLE* (*NZZ*)	It has a role in the establishment of the pollen sac and nucellus and possibly an early role in sporogenesis.	[59]
17	*NUBBIN* (*NUB*)	Define stamen and carpel shape. *NUB* acts redundantly with *JAG* to promote the growth of the pollen-bearing microsporangia of the anthers and the carpel walls of the gynoecium, which enclose the ovules. *JAG* and *NUB* also act redundantly to promote the differentiation of adaxial cell types in the carpel walls, and in the establishment of the correct number of cell layers.	[60]
18	*PISTILLATA* (*PI*) /*GLOBOSA* (*GLO*)	It acts with CLASS B MADS box gene *AP3*. (CLASS B MADS box gene)	[61]
19	*RABBIT EARS* (*RBE*)	Regulates the petal development by maintaining spatial boundries within young flowers	[62, 63]
21	*SPOROCYTELESS* (*SPL*)	It is required for the initiation of sporogenesis in male and female organs of the plants.	[64]
22	*SUPPRESSOR OF OVEREXPRESSION OF CONSTANS 1* (*SOC1*)	Integrates vernalization and gibberellin signals in *Arabidopsis*	[65]
23	*SHORT VEGETATIVE PHASE* (*SVP*)	*SVP* mediates the temperature-dependent functions of *FCA* and *FVE* within the thermosensory pathway. SVP controls flowering time by negatively regulating the expression of a floral integrator, *FLOWERING LOCUS T* (*FT*), via direct binding to the CArG motifs in the *FT* sequence.	[66]
24	*SUPERMAN* (*SUP*)	It is involved in controlling cell proliferation in stamen and carpel primordia and in ovules in flower development.	[67–71]
25	*TERMINAL FLOWER 1* (*TFL 1*)	It is putative regulator gene involved in the control of flowering time and floral architecture	[72, 73]
26	*WUSCHEL* (*WUS*)	WUS promotes central identity in both indeterminate shoot and determinate floral meristems and plays an important role in maintaining their structural and functional integrity.	[74]
27	*YABBY* (*YAB*)	Floral organ polarity	[54]
28	*SEPTALATA* (*SEP*)(*SEP1*, *SEP2*, *SEP3*, *SEP4*)	MADS box CLASS E genes. Role in ovule formation, required to specify petals, stamens and carpels	[45]
29	*FLOWERING LOCUS C* (*FLC*)	Delays flowering in plants. Represses FLOWERING TIME (FT) gene in the absence of low temperature/ vernalization treatment.	[58]
30	*FLOWERING LOCUS D* (*FLD*)	It encodes a plant homolouge of a protein found in histone deacetylase complexes in mammals. Lesions in FLD result in hyperacetylation of histones in FLC chromatin, up-regulation of FLC expression, and extremely delayed flowering.	[75]
31	*FLOWERING LOCUS T* (*FLT*)	It acts in parallel with the meristem identity gene LEAFY (LFY) to induce flowering of Arabidopsis.	[76]
32	*FRIGADIA* (*FRI*)	Delays flowering in plants. Promotes the expression of FLOWERING LOCUS C (FLC) in the absence of vernalization / low temperature.	[58]
33	*GIGANTIA* (*GI*)	Control of Flowering time in response to day length	[58]
34	*PHYTOCHROME A* (*PHYA*)	Far red light absorbing receptor gene which senses daylight changes to promote flowering. *Arabidopsis thalianaPHYA*-null mutantplants are insensitive to floral induction by day-length extensions or night-break light treatments for short-day–grown plants, both of which mimic long-day growth conditions.Under long-day growth conditions, *PHYA*-null mutant plants display a late-flowering phenotype when compared with the wild type plants.	[77–80]
35	*PHYTOCHROME B* (*PHYB*)	Far red light absorbing receptor gene. Itinhibits flowering in *Arabidopsis*. Loss of *PHYB* accelerates flowering under both long- and short-day conditions.	[81]
36	*SHORT INTEGUMENTS* (*SI*)	Controls ovule development and flowering time in *Arabidopsis*.	[82]
37	*FLOWERING PROMOTER FACTOR 1* (*FPF1*)	It is expressed after photoperiodic induction of flowering in A. thaliana. It is involved in GA-dependent signalling pathway and modulates a GA response in apical meristems during the transition to flowering.	[83]
38	*UNSUSUAL FLORAL ORGANS* (*UFO*)	Mediator between floral meristem identity genes and floral organ genes.	[84]
39	*FIMBRIATA* (*FIM*)	It mediates between floral meristem identity and floral organ genes. Expression and function of *FIM* depends on the activity of meristem identity genes, and *FIM* in turn controls the spatial and temporal expression of organ identity genes.	[85]
40	*ERECTA* (*ER*)	It encodes a putative receptor protein kinase. It regulates he shape of organs originating from the shoot apical meristem.	[86]
41	*DEFFECTIVE IN ANTHER DEHISCENCE 1* (*DAD1*)	It encodes chloroplastic phospholipase A1 that catalyzes the initial step of JA biosynthesis which synchronizes pollen maturation, anther dehiscence, and flower opening in *Arabidopsis*.	[87]
42	*ETHYLENE RESPONSE SENSOR 1* (*ERS*)	Ethylene receptor genes	[88]
43	*ETHYLENE RECEPTOR 1* (*ETR1*)	Ethylene receptor genes	[88]
44	*NO EXINE FORMATION 1* (*NEF1*)	Required in exine formation of pollen wall	[89]

**Table 2:** List of potential Seabuckthorn GISD retrieved from available seabuckthorn resources.

S. no.	Gene name	Contig No.^*^	Protein family, Domains & Repeats	Origin of Reference Genes	Identity	E-value	Accession no. of Reference Genes
1	*HrAP1*	87601	MADS box, K-box domain	*R. hybrid^*	76%	7e-67	FJ970028.1
				*A*. *thaliana*	-	-	-
2	*HrAP2*	31712	DNA binding domain, AP2/ERF domain	*V. vinifera^*	62%	3e-96	NP_001267881.1
				*A*. *thaliana*	78%	5e-55	NP_195410.1
3	*HrCLV1*	30543	Protein Kinase domain, Leucine rich repeats	*M. notabolis^*	76%	0.0	EXC25022.1
				*A*.*thaliana*	69%	0.0	AAB58929.1
4	*HrFLD*	20188	SWIRM, NADP, amine oxidase domain	*P. mume^*	85%	0.0	XP_008233274.1
				*A*.*thaliana*	81%	0.0	NP_187650.4
5	*HrCO*	32194	Zinc Finger B-box, CCT domain	*P. deltoids^*	72%	8e-178	AAS00054.1
				*A*.*thaliana*	54%	1e-114	NP_197088.1
6	*HrCOLK4*	24698	Zinc Finger B-box, CCT domain	*P. mume^*	74%	1e-167	XP_008220621.1
				*A*.*thaliana*	63%	2e-140	Q940T9.2
7	*HrCOLK9*	13913	Zinc Finger B-box, CCT domain	*F. vesca^*	68%	0.0	XP_004303586.1
				*A*.*thaliana*	56%	2e-139	NP_187422.1
8	*HrCRY1*	12695	Rossmann-like alpha/beta/alpha sandwich fold, DNA photolyase, N-terminal, DNA photolyase, FAD-binding/Cryptochrome, C-terminal, Cryptochrome C-terminal	*Populus trichocarpa^*	82%	0.0	XP_002307379.1
				*A*.*thaliana*	76%	0.0	NP_567341.1
9	*HrCRY1LK*	12696	Rossmann-like alpha/beta/alpha sandwich fold, DNA photolyase, N-terminal, DNA photolyase, FAD-binding/Cryptochrome, C-terminal, Cryptochrome C-terminal	*Populus trichocarpa^*	82%	0.0	XP_002307379.1
				*A*.*thaliana*	78%	0.0	NP_567341.1
10	*HrCRY2*	7867	Rossmann-like alpha/beta/alpha sandwich fold, DNA photolyase, N-terminal, DNA photolyase, FAD-binding/Cryptochrome, C-terminal	*Theobroma cacao^*	74%	0.0	XP_007035111.1
				*A*.*thaliana*	68%	0.0	NP_171935.1
11	*HrEF1*	34677	Helicase/SANT-associated, HAS subgroup	*G. max^*	51%	1e-88	XP_003518059.1
				*A*.*thaliana*	64%	7e-62	NP_187887.3
12	*HrEF3*	30075	N.D.	*Citrus sinensis^*	46%	0.0	XP_006466166.1
				*A*.*thaliana*	-	-	-
13	*HrFIL*	7258	YABBY protein, High mobility group box domain	*V. vinifera^*	80%	7e-84	XP_002266233.1
				*A*.*thaliana*	55%	7e-48	NP_566037.1
14	*HrFRI*	20160	Frgadia protein family	*V. vinifera^*	72%	0.0	XP_002282465.1
				*A*.*thaliana*	61%	0.0	NP_566709.1
15	*HrFRILK*	84388	Frgadia protein family	*V. vinifera^*	77%	0.0	XP_002266233.1
				*A*.*thaliana*	69%	0.0	NP_566709.1
16	*HrGI*	30943	N.D.	*P. mume^*	83%	0.0	XP_008237480.1
				*A*.*thaliana*	77%	0.0	ABP96488.1
17	*HrPHYB*	1355	PHY A/B/C/D/E protein family, PAF, GAF domain	*V. vinifera^*	85%	0.00	XP_002278263.1
				*A*.*thaliana*	-	-	-
18	*HrSI*	20174	P-loop, helicase, Dicer, Ribonuclease III, PAZ, DS RNA binding domain	*V. vinifera^*	87%	0.0	XP_002268369.1
				*A*.*thaliana*	80%	0.0	NP_171612.1
19	*HrTFL1*	8067	PEBP superfamily	*Citrus trifoliate^*	87%	8e-108	ABY91243.1
				*A*.*thaliana*	-	-	-
20	*HrNEF1*	18354	N.D.	*Theobroma cacao^*	84%	9e-87	XP_007043754.1
				*A*.*thaliana*	72%	1e-76	NP_196843.1
21	*HrSOC1*	8883	MADS box, K-box domain	*V. vinifera^*	71%	1e-98	ABF56527.1
				*A*.*thaliana*	-	-	-
22	*HrYAB5a*	21143	YABBY protein superfamily, HMG domain	*P. trichocarpa^*	78%	4e-73	XP_002308074.1
				*A*.*thaliana*	70%	1e-56	NP_850080.1
23	*HrYAB5b*	70948	YABBY protein superfamily, HMG domain	*P. mume^*	81%	5e-107	XP_008242786.1
				*A*.*thaliana*	72%	1e-77	NP_850080.1
24	*HrYAB4*	7257	YABBY protein superfamily, HMG domain	*G. max^*	82%	3e-117	XP_003549900.1
				*A*.*thaliana*	59%	2e-72	NP_566037.1
25	*HrSEP3*	15336	MADS box, K-box domain	*Shorea beccariana*	84%	1e-142	BAN89460.1
				*A*.*thaliana*	-	-	-
26	*HrACC*	8293	PPD transferase, Amino transferase CLASS I/II domain	*M. notabilis^*	80%	0.0	EXB37292.1
				*A*.*thaliana*	-	-	-
27	*HrETR1*	23688	Signal transduction histidine kinase, GAF domain	*P. domestica^*	86%	0.0	CAI64505.1
				*A*.*thaliana*	80%	0.0	NP_176808.3
28	*HrERS*	11717	Signal transduction histidine kinase, GAF domain	*T. cacao^*	82%	0.0	XP_007051012.1
				*A*.*thaliana*	-	-	-
29	*HrX1* ^*#*^	27099	AMP-dependent synthatase / ligase, AMP-binding enzyme C-terminal domain	*T. cacao^*	77%	0.0	XP_007034413.1
				*A*.*thaliana*	69%	0.0	NP_197138.1

^*^Contigs were obtained from the assembled unigenes of leaf and root transcriptome of *H*. *rhamnoides*[35].

Sequences of unigenes could be downloaded from http://www.plosone.org/article/fetchSingleRepresentation.action?uri=info:doi/10.1371/journal.pone.0072516.s004. For sequence analysis the nucleotide sequence was translated to protein sequence using Expasy translate

^#^Sequence of *HrX1* was obtained from Chawla et al, 2014 (NCBI Accession No.KF359497).

^ Plant species with maximum identity and minimum E-value.

### Expression analysis of GISD by qRT-PCR

Primers for candidate genes were designed using the Primer3 web application (http://bioinfo.ut.ee/primer3-0.4.0/), with Tm of 55–60°C and amplicon size between 100 bp and 250 bp (S1 Table). qRT-PCR was performed with duplicate amplifications using SYBR-green-based detection system (IQ SYBR Green Supermix (Biorad) in the Biorad CFX96 Real-Time PCR Detection System). The reactions contained 100 ng cDNA template and 0.5μM of primers in total volume of 13μl. Cycle parameters of reaction were 95°C for 3 min and then 39 cycles at 95°C for 10, 60°C for 30 s and 72 for 20s. Expression data were analysed with ΔΔCT method [41]. The expression of four internal reference genes namely ubiquitin, β-actin, 26S and GAPDH was checked on four floral bud samples. 26S and GAPDH genes showed consistent expression pattern in male and female flower bud stages (Unpublished data) and were used for gene expression data normalisation. The data presented in the figures and tables are based on the average of 2 PCR samples used from 3 biological samples. Fold expression of genes was calculated between the same development stages of male and female flowers. Heat map representing the gene expression data of GISD in three developmental stages of male and female seabuckthorn flowers was generated using the GENEX Ver. 6.0 software (http://genex.gene-quantification.info).

## Results

### Identification of seabuckthorn homologues of potential GISD and phylogenetic analysis

**Fig 2:**
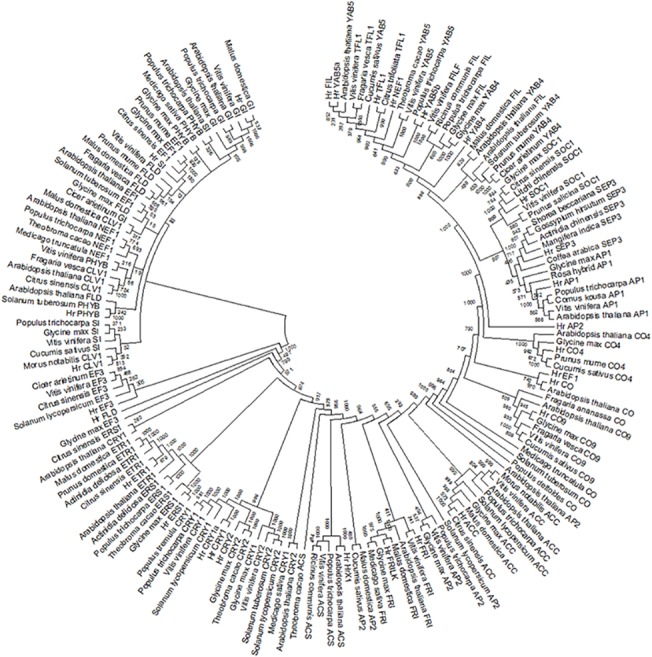
Phylogenetic tree of the potential GISD in Seabuckthorn (*H*. *rhamnoides*) based on the amino acid sequence alignment.

The current study was focused on 44 *Arabidopsis* genes that were known to be involved in floral regulatory pathways (Table 1) and could be probable candidates for sex determination in *H*. *rhamnoides*. Out of 44 *Arabidopsis* flowering genes, 24 genes had homologous sequences in available *H*. *rhamnoides* genomic resources (Table 2). Arabidopsis genes for which homologous sequences were not present in the transcriptome data of seabuckthorn include *AP3*, *CAL*, *CRC*, *JAG*, *KNU*, *LFY*, *NZZ*, *NUB*, *RBE*, *SPL*, *SVP*, *SUP*, *WUS*, *FLC*, *FLT*, *UFO*, *FIM*, *ER* and *DAD1*. The identified homologous sequences of seabuckthorn GISD were compared with similar genes of other plants species deposited in NCBI genebank nucleotide database as well as EST databases of other plant species like *Actnidia chinesis* [90]. Results of the analysis showed that the sequences of putative seabuckthorn GISD matched with transcripts of either one plant species or the other (Table 2). Also more than one copy of homologous sequences were found for genes *CO* (3), *CRY1* (2), *FRI* (2) and *TFL* (2) in *H*. *rhamnoides* (Table 2). Domains and repeats found in all the homologues of seabuckthorn except for *EF3*, *GI* and *NEF1* were similar to those present in *Arabidopsis* genes (Table 2). Such an outcome signifies that the identified contigs of putative seabuckthorn GISD have similar gene structure as of *A*. *thaliana* genes and thus are likely to perform identical functions as performed by respective genes in *A*. *thaliana*. Phylogenetic reconstruction of genes (Fig 2) showed that most of the seabuckthorn GISD clustered with similar genes except for *HrCRY2*, *HrFIL*, *HrAP2* and *HrNEF*. Clustering of putative seabuckthorn GISD along with characterized genes in model plants further confirms that putative seabuckthorn genes share high homology to well characterized genes in model plants.

### Expression analysis of GISD by qRT PCR

**Fig 3:**
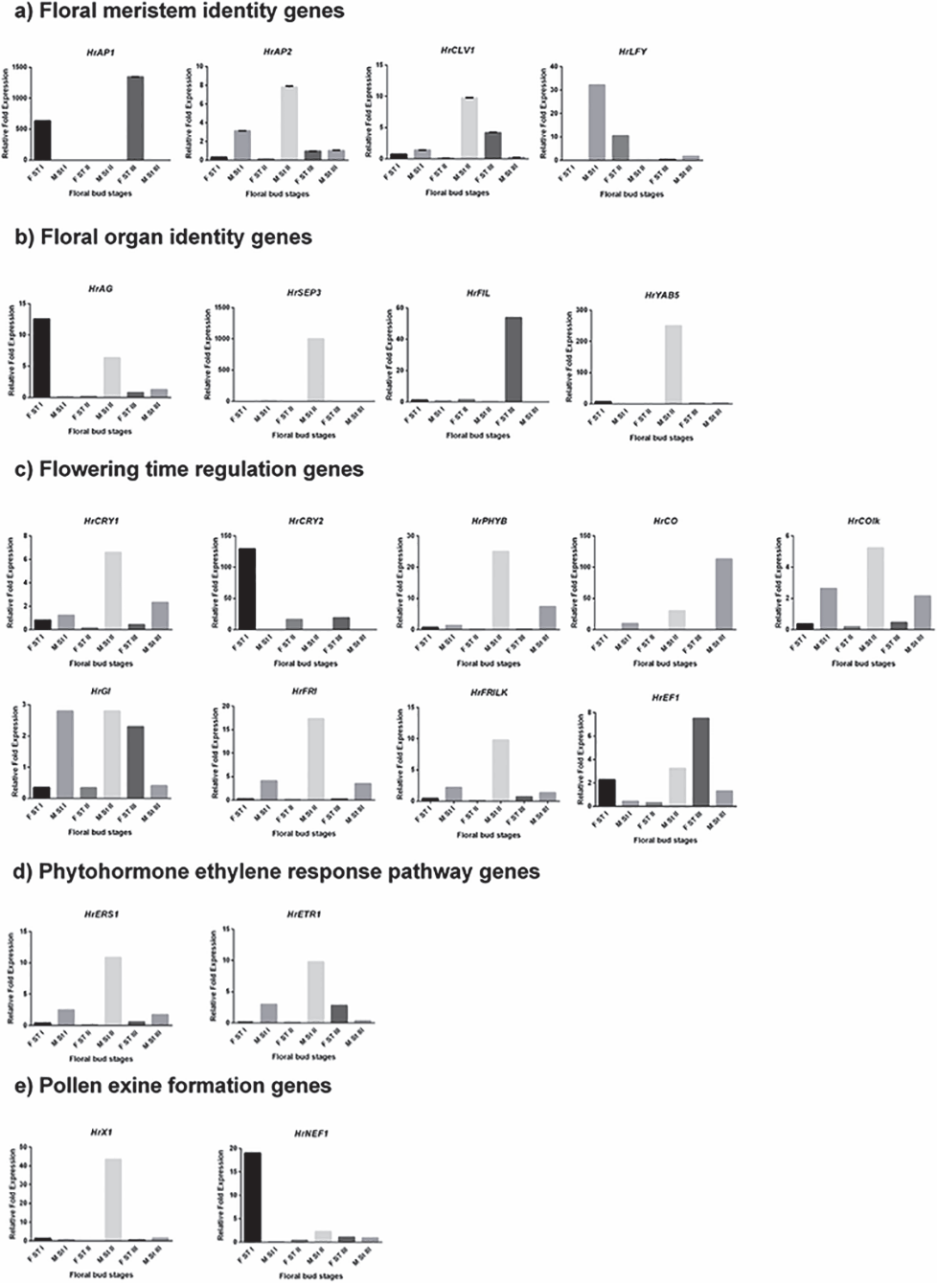
Relative expression of Putative GISD.

The temporal expression of the 24 potential GISD and their additional homologues (Total of 29 candidate GISD) across three developmental stages of male and female flowers of *H*. *rhamnoides* was analysed (S2 Table). 21 GISD were analysed for differential expression among temporally corresponding male and female flower developmental stages (FDS). The CT values of eight GISD which showed values greater than 35, were not considered for further investigation. Seven GISD showed elevated expression in female FDS while fourteen GISD showed higher expression in male FDS, (Fig 3 and Table 3) details of which are given below.

**Table 3:** Relative fold expression of putative GISD within temporally corresponding male and female flower development stages.

Genes	FST I	MST I	FST II	MST II	FST III	MST III
***HrAP1***	**632**	0.002	0.84	1.18	**1347**	N.E. *
***HrAP2***	0.32	**3.14**	0.13	**7.70**	0.97	**1.02**
***HrCLV1***	0.72	1.39	0.10	**9.71**	**4.15**	0.24
***HrLFY***	0.031	**32.16**	**10.51**	0.095	0.56	1.70
***HrAG***	**12.55**	0.08	0.16	**6.34**	0.79	1.27
***HrSEP3***	0.77	1.29	0.001	**1000**	0.87	1.14
***HrFIL***	1.22	0.82	1.62	0.62	**53.88**	0.02
***HrYAB5***	**7.62**	0.13	0.004	**250**	0.57	1.73
***HrCRY1***	0.81	1.23	0.15	**6.6**	0.43	2.33
***HrCRY2***	**129.3**	0.008	**16.41**	0.060	**19.19**	0.052
***HrPHYB***	0.74	1.35	0.04	**25**	0.13	**7.4**
***HrCO***	0.100	**9.91**	0.033	**30.30**	0.009	**113.14**
***HrCOLK***	0.37	2.64	0.191	**5.23**	0.46	2.15
***HrGI***	0.35	**2.8**	0.34	**2.8**	**2.3**	0.41
***HrFRI***	0.24	**4.1**	0.06	**17.31**	0.28	**3.5**
***HrFRILK***	0.45	2.18	0.108	**9.78**	0.72	1.37
***HrEF1***	2.27	0.44	0.31	3.25	**7.52**	1.32
***HrERS1***	0.40	2.50	0.092	**10.86**	0.58	1.73
***HrETR1***	0.34	2.98	0.108	**9.78**	2.8	0.36
***HrX1***	1.34	0.74	0.02	**43.47**	0.65	1.54
***HrNEF1***	**19.01**	0.05	0.42	2.3	1.09	0.91

* Fold expression value less than 0.0001 are considered as Negligible Expression (N.E.).

#### Floral meristem identity genes

As data presented in Table 3 and Fig 3A demonstrates, meristem identity gene *HrAP1* showed female specific expression. The expression of this gene was notably 1347 fold higher in FST II as compared to MST II. On the contrary, expression of gene *HrAP2* was higher in all the male flower developmental stages withthe maximum differential expression being in MST II (7.70 fold) as compared to FST II. *HrLFY* and *HrCLV1* showed stage specific expression in male and female FDS. The expression of *HrLFY* was notably higher in MST I (32.16 fold) and FST II (10.15 fold) as compared to their corresponding stages. *HrCLV1* was significantly expressed in MST II (9.11 fold) and FST III (4.15 fold). On the basis of this data it is concluded that expression of gene *HrAP1* is female specific while that of *HrAP2* is male specific. However, the expression of gene *HrLFY* and *HrCLV1* in male and female flowers was stage dependent.

#### Floral organ identity genes

Among floral organ identity genes the expression of floral organ polarity gene *HrFIL* was higher in all FDS of female flowers (Fig 3B). The differential expression was notably wider in FST III vs MST III (53.88 fold). On the contrary the expression of *HrYAB5* and *HrSEP3* was higher in all male FDS with highest differential expression of 250 fold and 1000 fold were recorded in MST II respectively. Stamen and carpel identity gene AGAMOUS (*HrAG*) showed stage dependent expression pattern which was higher in FST I (12.55 fold) and MST II (6.34 fold) as compared to their corresponding stages. Thus from the data recorded it can be concluded that expression of floral organ identity gene *HrSEP3* and *HrYAB5* was higher in male FDS and that of *HrFIL* was higher in female FDS. Also the relative expression of gene *HrAG* was flower developmental stage dependent rather than sex of flower.

#### Flowering time regulation genes

The expression of Blue-Ultraviolet A receptor gene *CRYPTOCHROME2* (*HrCRY2*) was higher across all the female FDS as compared to male FDS. The expression of this gene was 129.3 fold higher. *CRYPTOCHROME1*(*HrCRY1*) was relatively expressed higher in all male FDS with MST II and MST III showing 6.6 fold and 2.33 fold higher expression as compared to corresponding female FDS (Fig 3C). Similarly the expression of far red light receptor gene *PHYTOCHROME B* (*HrPHYB*) was higher in all male FDS notably MST II and MST III, which showed 25 fold and 7.5 fold higher expression with respect to FST II and FST III respectively (Fig 3C). The expression of *CONSTANS* (*HrCO*) responsible for flowering in long days was higher in all male FDS (Fig 3C) (9.91 fold in MST I with respect to FST I, 30 fold in MST II with respect to FST II and 113 fold in MST III with respect to FST III). The second homologue of *CO* (*HrCOLK*) showed similar pattern of expression but relative difference in a expression level was less pronounced in male and female FDS as compared to *HrCO*. *FRIGADIA* (*HrFRI*) and its second hoologue *HrFRILK* responsible for delayed flowering in absence of cold temperatures, were also found to have elevated expression in male FDS as compared to their corresponding female FDS (*HrFRI* 17.31 fold higher in MST II; *HrFRILK* 9.78 fold higher in MST II) (Fig 3C). The relative expression of genes *HrGI* and *HrEF1* was stage dependent. Thus it is concluded that expression of most of flowering time genes including *HrCRY1*, *HrPHYB* and *HrCO* was higher in all male FDS while that of *HrCRY2* was higher in all female FDS.

#### Phytohormone ethylene response pathway genes

The expression of seabuckthorn homologues of ethylene response pathway genes *ETHYLENE RESPONSE SENSOR 1* (*HrERS1*) and *ETHYLENE RECEPTOR 1* (*HrETR1*) was higher in male flowers. *HrERS1* (Fig 3D) showed 10.86 fold higher expression in MST II with respect to FST II while expression of *HrETR1* (Fig 3D) was recorded 9.78 fold higher in MST II with respect to FST II.

#### Pollen exine formation genes

*HrX1* is the female specific SCAR marker which was found to show high level of similarity to Acyl CoA synthatase and other related plant ligases on the basis of BLASTn and tBLASTx analysis of sequence [8]. *HrX1* (Fig 3E) expressed 43.47 fold higher in MST II with respect to FSTII. On the contrary, expression of *HrNEF1* (Fig 3E) was observed to be 19.01 fold higher in FST I as compared to MST I.

### Floral development stage (FDS) specific expression of GISD

**Fig 4:**
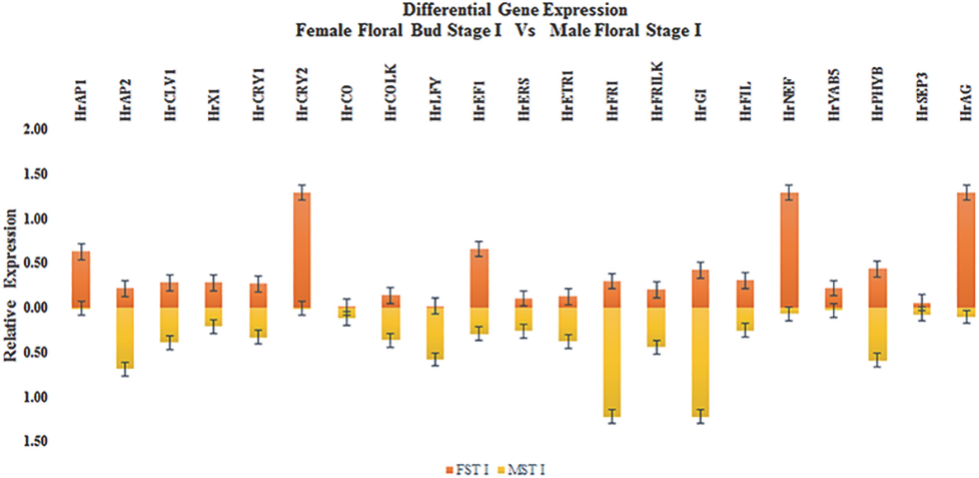
Comparative expression of seabuckthorn GISD between floral Development Stages (FDS)—Male stage I (MST I) vs Female Stage I (FST I).

**Fig 5:**
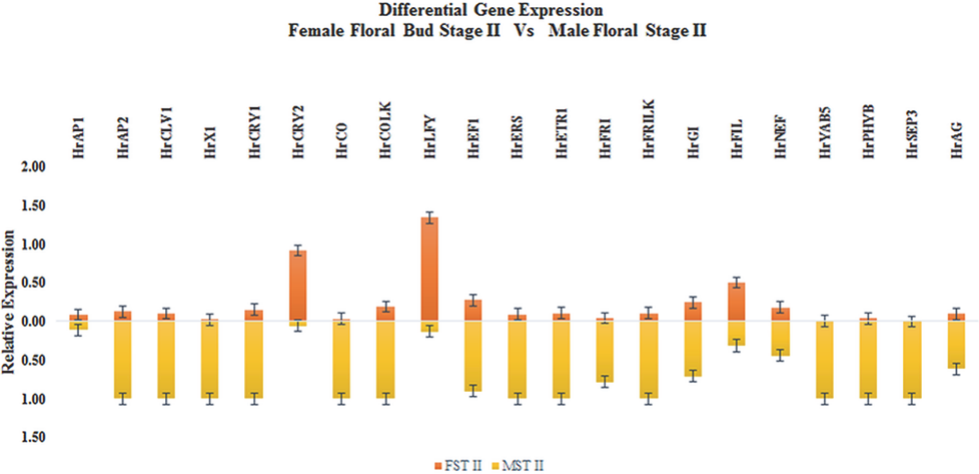
Comparative expression of seabuckthorn GISD between floral Development Stages (FDS)—Male stage II (MST II) vs Female Stage II (FST II).

**Fig 6:**
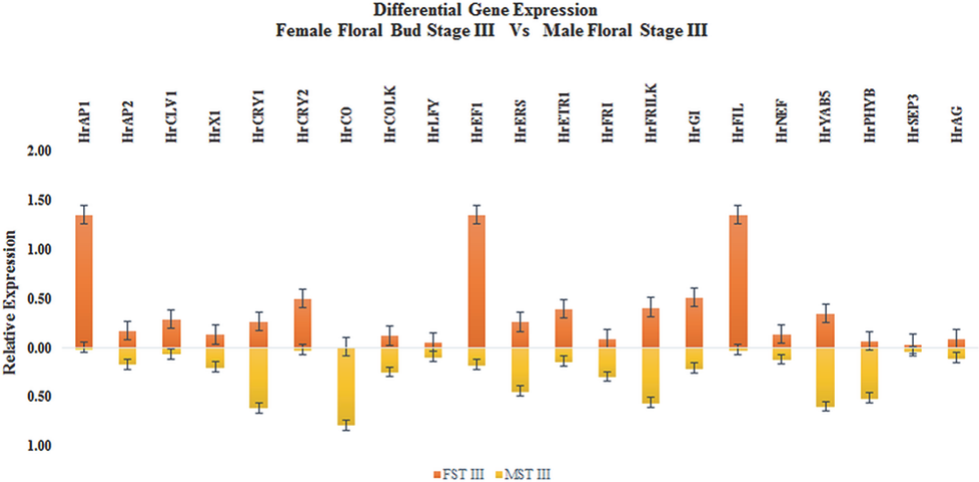
Comparative expression of seabuckthorn GISD between floral Development Stages (FDS)—Male stage III (MST III) vs Female Stage III (FST III).

**Fig 7:**
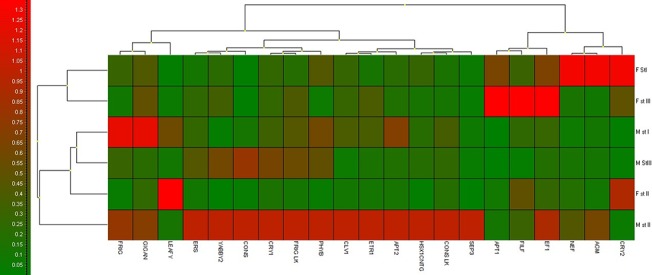
Heat map of relative expression of putative GISD in seabuckthorn FDS.

In stage I of flower development the expression of genes *HrAP1*, *HrCRY2*, *HrEF1*, *HrNEF* and *HrAG* was higher in female flowers while expression of genes *HrAP2*, *HrLFY*, *HrFRI*, and *HrGI* was higher in male flowers (Fig 4). The expression of all putative GISD except for *HrCRY2* and *HrLFY* was higher in 2^nd^ developmental stage of male flowers (Fig 5). In STAGE III FDS female flowers had higher expression of *HrAP1*, *HrCRY2*, *HrEF1* and *HrFILF* while male flowers had higher expression levels of *HrCRY1*, *HrCO* and *HrPHYB* (Fig 6). Moreover, the heat map of putative GISD (Fig 7) shows that male flowers have maximum GISD with higher level of expression as compared to female flowers in 2^nd^ floral developmental stage.

#### Discussion

The identified putative GISD of seabuckthorn shared the sequence similarity with plant species like *R*. *hybrid*, *V*. *vinefera*, *M*. *notabilis*, *P*. *trichocarpa*, etc. and had similar repeats and domains as *Arabidopsis* floral regulatory genes. Most of these genes also clustered along with well characterised genes of model plants. Identification of homologous flowering genes in seabuckthorn reflects that flowering pathways of seabuckthorn share similarity with *Arabidopsis* as well as other model dioecious plants. Thus as in the case of *S*. *latifolia*, *R*. *acetosa* and *A*. *chinensis*, genes involved in these flowering pathways could be potential candidates of sex determination in seabuckthorn.

Expression pattern of MADS box genes in male and female flowers of sorrel (*Rumex acetosa*) suggested that these genes could play an important role in sex determination [31]. CLASS A MADS box gene *HrAP1* (Fig 3A) showed female specific expression while *HrAP2* (Fig 3A), expressed particularly in male flowers (Fig 3A). *AP2* plays an important role for sex determination in maize [35]. It suppresses carpel in tassel of male flowers by targeting *TASSELSEED 4* (*TSL4*). Similarly expression of another floral meristem identity gene *HrCLV1* (Fig 3A) was recorded highest in MST II. In case of *S*. *latifolia*, *CLV1* triggers carpel suppression in male flowers [36]. Thus *HrAP2* and *HrCLV1* may be involved in determining meristem identity in male flowers while *HrAP1* could trigger meristem development in female flowers of seabuckthorn.

Expression of floral organ identity gene *HrSEP3* (Fig 3B) was recorded highest in MST II. Higher expression of *SEP3* homologue was also observed in male flowers of *Asparagus officinalis* [34]. On the other hand *HrAG* (Fig 3B) showed significant expression in FST II. Thus *HrSEP3* and *HrAG* may have a crucial role in establishing floral organ identity in male and female flowers respectively.

The expression of cryptochrome receptor gene *HrCRY2* (Fig 3C) was higher in female flowers as compared to male flowers. On the other hand, level of expression of cryptochrome gene *HrCRY1* (Fig 3C), phytochrome gene *HrPHYB* (Fig 3C) and circardian pathway gene *HrCO* (Fig 3C) was higher in all male flower development stages. In dioecious plants like *S*. *latifolia* and *Populus tomentosa*, male and female flowers develop at different time. The photoreceptor encoding genes like *CRY1*, *CRY2*, *PHYA* and *PHYB* regulate circardian pathway genes like *CO*, *GI* and *FT* and could alter flowering time depending upon external cues [91]. Differential expression of *CRY1*, *CRY2*, *CO* and *GI* was observed among male and female flowers of *P*. *tomentosa* and was correlated with asynchronous development of male and female flowers [92]. Thus expression pattern of flowering time genes showed that *HrCRY2* could influence time-dependent development of female flowers while *HrCRY1*, *HrPHYB* and *HrCO* may affect temporal development of male flowers in seabuckthorn.

Phytohormone ethylene response genes *HrERS1* and *HrETR1* differentially expressed in all the stages of male and female flower but without bias of expression towards particular gender. Such an outcome could be expected because, in case of dioecious plants genetic variations have a more prominent role in gender determination than internal environment and environment variation. Expression of gene containing the female specific SCAR marker *HrX1* was higher in male flowers as compared to female flowers. *HrX1* shares sequence similarity with known plant ligases such as acyl Coa synthatase [8]. In *A*. *thaliana* knocking out of acyl CoA synthatase led to production of unviable pollen, which in turn produced male sterile plants [93]. Another pollen exine gene *HrNEF1* showed higher expression in female flowers as compared to male flowers. Disruption of *NEF1* in *A*. *thaliana* affected lipid accumulation in the plastids of tapetum as well as exine formation of pollen, thus resulted in male sterility in *A*. *thaliana* [89]. Thus expression pattern of *HrX1* and *HrNEF1* suggested that these genes could play an important in sex determination in seabuckthorn.

The expression of genes varied throughout the development of flowers in both male and female flowers of seabuckthorn. Out of the three developmental stages, 2^nd^ stage had the maximum number of genes with expression biased towards male flowers (Fig 4 and Fig 5 & Fig 6). Thus stage II of male and female flowers require further investigation to justify the tilt of GISD expression towards male flowers.

In conclusion, the current study showed differential expression of putative seabuckthorn GISD in all the three floral developmental stages of both male and female flowers. The expression level of *HrCO* gene was observed higher in the developmental stages of male flowers as compared to female flowers. Whereas *HrCRY2* gene significantly showed higher expression levels in the female floral developmental stages only. Further investigation is required to understand the role of *HrCO* and *HrCRY2* genes in development of male and female flowers respectively.

## Supporting Information

S1 File Nucleotide sequences of the known flowering pathway genes in model plants. (RAR)

S2 File Nucleotide sequences of seabuckthorn putative GISD retrieved from seabuckthorn genomic resources. (TXT)

S3 File Protein sequences of seabuckthorn putative GISD. (TXT)

S4 File Protein sequences of the known flowering pathway genes in model plants. (TXT)

S1 Table List of primers used in qRT PCR analysis of putative GISD. (DOCX)

S2 Table Normalized expression values of seabuckthorn putative GISD in three temporal developmental stages of male and female flowers. (DOCX)
