# Supplementary material for: Sex-Biased Temporal Gene Expression in Male and Female Floral Buds of Seabuckthorn (Hippophae rhamnoides)
Source: PLoS One. 2015 Apr 27;10(4):e0124890. doi: 10.1371/journal.pone.0124890 (PMC4410991; doi:10.1371/journal.pone.0124890)
Supplement: S1 Table — (DOCX) [file pone.0124890.s005.docx]

**Supporting Information Table S1 – List of primers used in qRT PCR analysis of putative GISD.**

| **S.No.** | **Primer Name** | **Primer Sequence** | **Length** |
| --- | --- | --- | --- |
| **1** | HSX1CNTG1 F | CGTCGTTCCTGAGGTGTAGT | 20 |
| **2** | HSX1CNTG1 R | GCTCTCCCTCTTTCCTCCTC | 20 |
| 3 | HSX1CNTG2 F | TCACCATCGGATCCCATTCA | 20 |
| 4 | HSX1CNTG2 R | TTGAAGCGCTCTCCCTCTTT | 20 |
| 5 | HSX1CNTG3 F | TTCATCACCATCGGATCCCA | 20 |
| 6 | HSX1CNTG3 R | CTATACTCCTCCGCCACAGC | 20 |
| 7 | HSX1CNTG4 F | TCGGAATGGAGAAGTCGACA | 20 |
| 8 | HSX1CNTG4 R | TTCTTGAAGCGCTCTCCCTC | 20 |
| 9 | RTFLD F | GTCAAAACACTCCCGCCTAA | 20 |
| 10 | RTFLD R | GCTTGGTGTGGTTGTGATTG | 20 |
| 11 | RTAGL15 F | TGCCTCTCTTTGCCAGAACT | 20 |
| 12 | RTAGL15 R | GTTCTCGAAGCGAAGGAATG | 20 |
| 13 | RTCONS F | ACATACTGGCCCGAATTGAG | 20 |
| 14 | RTCONS R | CGACAACGCCAACTCTAACA | 20 |
| 15 | RTCONSB F | CGGCATCGCTTTGTACTTCT | 20 |
| 16 | RTCONSB R | TCCCGTTCTTCACTGGATTC | 20 |
| 17 | RTCONS2 F | GAGCGTGTCTGGGTATGTGA | 20 |
| 18 | RTCONS2 R | ACGAACCCATCTTCAGCATC | 20 |
| 19 | RTCRY2 F | AGTTTCAAGGTGGTGGCTGA | 20 |
| 20 | RTCRY2 R | TGCACAGAAATGCCTAGCTC | 20 |
| 21 | RTCRY1 F | GGAGAGTCGAGCAGAAGTGC | 20 |
| 22 | RTCRY1 R | CTGTCGTGGAATCTTCAGCA | 20 |
| 23 | RTCRY1B F | CCGAATGGATACACCATCCT | 20 |
| 24 | RTCRY1B R | GTGGGAAGGCAATGACAGTT | 20 |
| 25 | RTEF1 F | GGCTCAGGCAAAGAAAGTTG | 20 |
| 26 | RTEF1 R | TCTCGTCAACCTCCATCTGA | 20 |
| 27 | RTFILF F | GTTCGATGTGGTCACTGCAC | 20 |
| 28 | RTFILF R | GCAAAGTCATGGAGGCTTGT | 20 |
| 29 | RTFRIG F | CACTTGTGGTTGATCCGTTG | 20 |
| 30 | RTFRIG R | CTGTTTCCTCCAAGCAGACC | 20 |
| 31 | RTFRIGLK F | GGTCGAACAGAGCAAAGAGG | 20 |
| 32 | RTFRIGLK R | CTAATGGCAACTGGGCTCTC | 20 |
| 33 | RTFRUITFL F | ATGGAGGTGATCCTTGAACG | 20 |
| 34 | RTFRUITFL R | CCAGTTCGTCTCCCTTGAAA | 20 |
| 35 | RTGIGAN F | GATGGGCTGTTGCTAATGGT | 20 |
| 36 | RTGIGAN F | TGTGTGGCACTTGGAGTAGC | 20 |
| 37 | RTPHYB F | GGTTCCTTCCACCAACAGAA | 20 |
| 38 | RTPHYB R | TGATGCAGCCTCTATGCTTG | 20 |
| 39 | RTSHTINTG F | CCATGCCTTCAGAAAGGGTA | 20 |
| 40 | RTSHTINTG R | CCTGCCTCAACTCTTCCTTG | 20 |
| 41 | RTTRMFLR F | TGGCCTTGGAATCTCATAGC | 20 |
| 42 | RTTRMFLR R | CTTCCACAGTCACCACCAAA | 20 |
| 43 | RTTRMFLRLK F | AAGGTCGAGCCACTGACTGT | 20 |
| 44 | RTTRMFLRLK F | TTCGGAAAAGAGGTGGTGAG | 20 |
| 45 | RTAPT2 F | CTGCTTCAATCTCGGTGTCA | 20 |
| 46 | RTAPT2 R | CGCCGACAAAGTACAGGATT | 20 |
| 47 | RTAPT1 F | ATGGGCCTGTATCTGAAACG | 20 |
| 48 | RTAPT1 R | CCAGTTCGTCTCCCTTGAAA | 20 |
| 49 | RTCLV1 F | GGTCGCATTCCAGAGTTCAT | 20 |
| 50 | RTCLV1 R | AAATCACGAGGCACAAGTCC | 20 |
| 51 | RTNEF1 F | GAGCTATCGTTGTGGGCTTC | 20 |
| 52 | RTNEF1 R | GCCAACACAGCACTAGCAAC | 20 |
| 53 | RTSOC1 F | CAAAACTTGCAGCACCTGAA | 20 |
| 54 | RTSOC1 R | GCTAGGGCCATTTCCTTTTC | 20 |
| 55 | RTYABBY1 F | CCTTTGGATGAAGAGCCTGA | 20 |
| 56 | RTYABBY1 R | TTGCGGTTAGTGTACCATGC | 20 |
| 57 | RTYABBY2 F | AAGGCTTCCCTGTGGCTAAT | 20 |
| 58 | RTYABBY2 R | TGACTCAGCCTCTTCATCCA | 20 |
| 59 | RTYABBY3 F | ACCTAACCCATCACCGAACA | 20 |
| 60 | RTYABBY3 R | CGTTGGATCTCGTCCTTGAT | 20 |
| 61 | RTSEP3 F | TTGTGATGCAGAGGTTGCTC | 20 |
| 62 | RTSEP3 R | GGCTAGCAAACTCCAATGCT | 20 |
| 63 | RTACS1F | GTTTGGCTGATCCTGGTGAT | 20 |
| 64 | RTACS1R | TCCCTAGTGGGTTTGATGGA | 20 |
| 65 | RTETR1 F | GTCCACTGCCACCAGAATTT | 20 |
| 66 | RTETR1 R | GTTCTCAAAAGAGGGCACCA | 20 |
| 67 | RTGAPDH F | AGGCCATCAAGGAGGAATCT | 20 |
| 68 | RTGAPDH R | AACTGTAGCCCCATTCGTTG | 20 |
| 69 | RTERS F | GGAGGAATGTGCCTTATGGA | 20 |
| 70 | RTERS R | ACCCGAACAGCAACAACTTC | 20 |
