# Supplementary material for: Sex-Biased Temporal Gene Expression in Male and Female Floral Buds of Seabuckthorn (Hippophae rhamnoides)
Source: PLoS One. 2015 Apr 27;10(4):e0124890. doi: 10.1371/journal.pone.0124890 (PMC4410991; doi:10.1371/journal.pone.0124890)
Supplement: S2 Table — (DOCX) [file pone.0124890.s006.docx]

**Supporting Information Table S2- Normalized expression values of seabuckthorn putative GISD in three temporal developmental stages of male and female flowers**

| **gene name** | **F StI** | **F st II** | **F st III** | **M st I** | **M st II** | **M StIII** |
| --- | --- | --- | --- | --- | --- | --- |
| **HrAP1** | 0.632 | 0.09 | 1.347 | 0.001 | 0.107 | 0.001 |
| **HrAP2** | 0.219 | 0.13 | 0.168 | 0.688 | 1 | 0.172 |
| **HrCLV1** | 0.281 | 0.103 | 0.287 | 0.391 | 1 | 0.069 |
| **HrX1** | 0.281 | 0.023 | 0.129 | 0.209 | 1 | 0.199 |
| **HrCRY1** | 0.267 | 0.151 | 0.264 | 0.33 | 1 | 0.617 |
| **HrCRY2** | 1.293 | 0.919 | 0.499 | 0.01 | 0.056 | 0.026 |
| **HrCO** | 0.012 | 0.033 | 0.007 | 0.119 | 1 | 0.792 |
| **HrCOLK** | 0.138 | 0.191 | 0.117 | 0.365 | 1 | 0.252 |
| **HrLFY** | 0.018 | 1.346 | 0.053 | 0.579 | 0.128 | 0.095 |
| **HrEF1** | 0.659 | 0.277 | 1.347 | 0.29 | 0.901 | 0.179 |
| **HrERS** | 0.105 | 0.092 | 0.257 | 0.263 | 1 | 0.445 |
| **HrETR1** | 0.127 | 0.108 | 0.392 | 0.379 | 1 | 0.14 |
| **HrFRI** | 0.298 | 0.045 | 0.084 | 1.223 | 0.779 | 0.297 |
| **HrFRILK** | 0.203 | 0.108 | 0.408 | 0.443 | 1 | 0.561 |
| **HrGI** | 0.423 | 0.247 | 0.51 | 1.222 | 0.707 | 0.213 |
| **HrFIL** | 0.307 | 0.505 | 1.347 | 0.251 | 0.312 | 0.025 |
| **HrNEF1** | 1.293 | 0.185 | 0.135 | 0.068 | 0.441 | 0.123 |
| **HrYAB5** | 0.221 | 0.004 | 0.347 | 0.029 | 1 | 0.601 |
| **HrPHYB** | 0.436 | 0.04 | 0.069 | 0.591 | 1 | 0.515 |
| **HrSEP3** | 0.055 | 0.001 | 0.035 | 0.071 | 1 | 0.04 |
| **HrAG** | 1.293 | 0.097 | 0.084 | 0.103 | 0.615 | 0.107 |
